# Transformation from lung adenocarcinoma to pulmonary giant cell carcinoma following targeted therapy: a case report

**DOI:** 10.3389/fonc.2026.1846681

**Published:** 2026-07-14

**Authors:** Shicheng Yuan, Qun Zhang, Yanling Xu, Yanfeng Wu, Qi Wang

**Affiliations:** 1 Department of the Second Clinical Medical College, Jilin University, Changchun, China; 2 Department of Respiratory and Critical Care Medicine, the Second Hospital of Jilin University, Changchun, China

**Keywords:** adenocarcinoma, giant cell carcinoma, KRAS, lung cancer, targeted therapy

## Abstract

Pathological subtype transformations may occur during the targeted therapy for lung adenocarcinoma. Among these, transformation to pulmonary giant cell carcinoma (PGCC) is rare, but clinically significant. This paper reports the case of a 71-year-old male patient with lung adenocarcinoma harboring the *KRAS G12C* mutation who developed resistance to sotorasib-targeted therapy, and subsequent re-biopsy confirmed transformation to PGCC. Combined with a literature review, the possible mechanisms of this transformation and its clinical implications were analyzed, emphasizing the importance of re-biopsy after targeted therapy resistance to guide subsequent treatment and provide a reference for the diagnosis and treatment of similar cases.

## 1 Introduction

Pulmonary giant cell carcinoma (PGCC) is a rare and highly malignant subtype of pulmonary sarcomatoid carcinoma that accounts for less than 1% of all lung cancers. It is characterized by highly atypical tumor cells and multinucleated giant cells, rapid growth, strong invasiveness, high early metastasis rate, and extremely poor prognosis (1). The *KRAS* mutation is a common driver gene in lung adenocarcinoma, and targeted therapy for the G12C mutant subtype has become the standard clinical regimen. However, the disease progression pattern and pathological transformation mechanisms after drug resistance require further exploration (2). Recently, our hospital admitted a patient with lung adenocarcinoma who developed PGCC after developing resistance to *KRAS G12C* inhibitor therapy. The diagnosis, treatment process, and transformation mechanism were analyzed and reported, combined with a literature review.

## 2 Case description

A 71-year-old married male admitted to the hospital with fever accompanied by cough and asthma for more than 10 days. Chest computed tomography (CT) revealed an enlarged space-occupying lesion in the right middle lobe with obstructive pneumonia.

### 2.1 Past history

**Figure 1:**
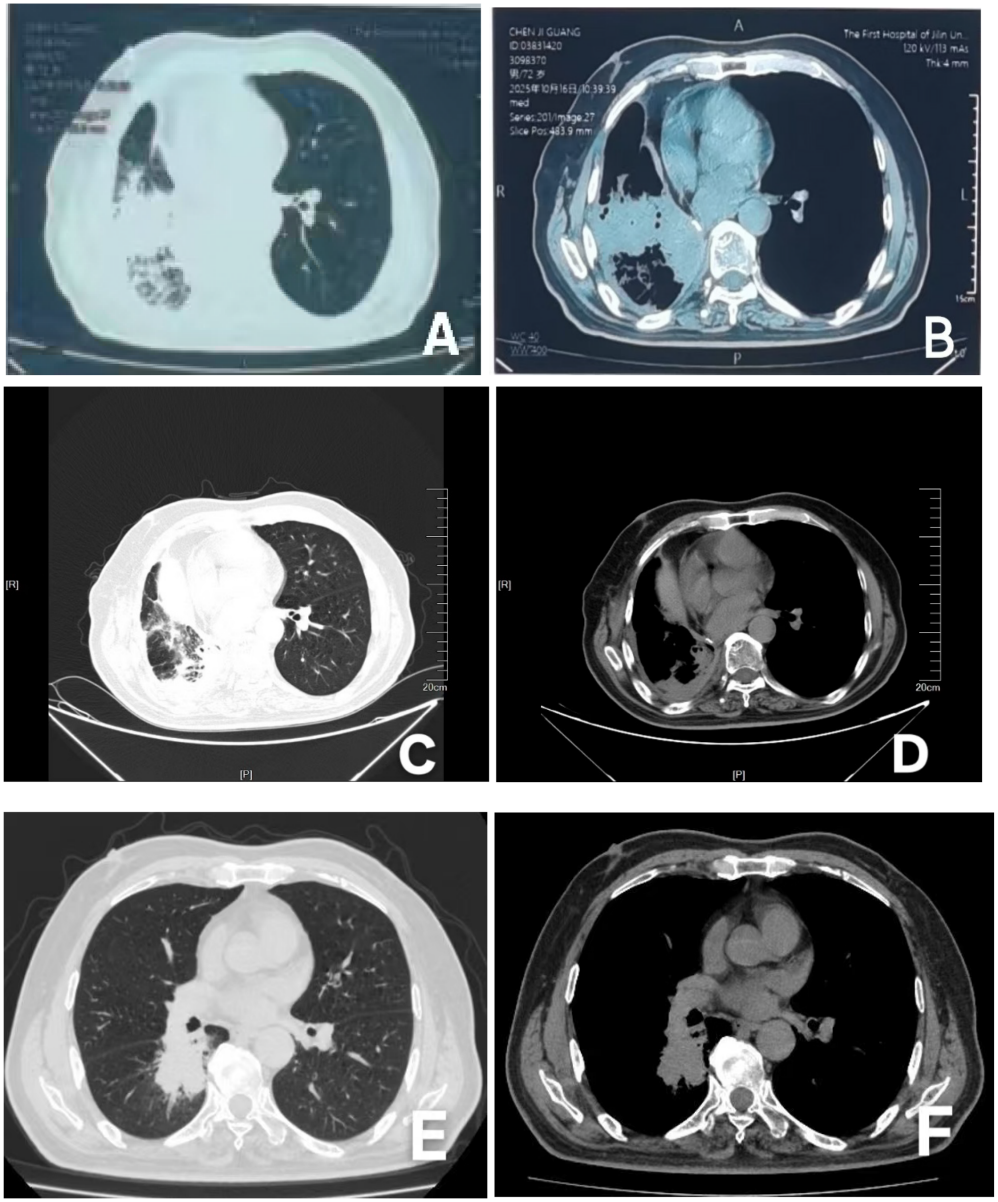
Chest CT imaging manifestations. (**(A)** Lung window at the initial diagnosis in 2026; mass in the right middle lobe. **(B)** Mediastinal window at initial diagnosis in 2026; mass in the right middle lobe. **(C)** Lung window at re-examination in 2026 after immunotherapy: postoperative changes in the right lung, multiple patchy high-density shadows in the residual right lung. **(D)** Mediastinal window at re-examination in 2026 after immunotherapy: postoperative changes in the right lung, multiple patchy high-density shadows in the residual right lung. **(E)** Lung window at the initial diagnosis in 2022; mass across the oblique fissure involving the posterior segment of the right upper lobe and the dorsal segment of the right lower lobe. **(F)** Mediastinal window at initial diagnosis in 2022; mass across the oblique fissure involving the posterior segment of the right upper lobe and the dorsal segment of the right lower lobe).

**Figure 2:**
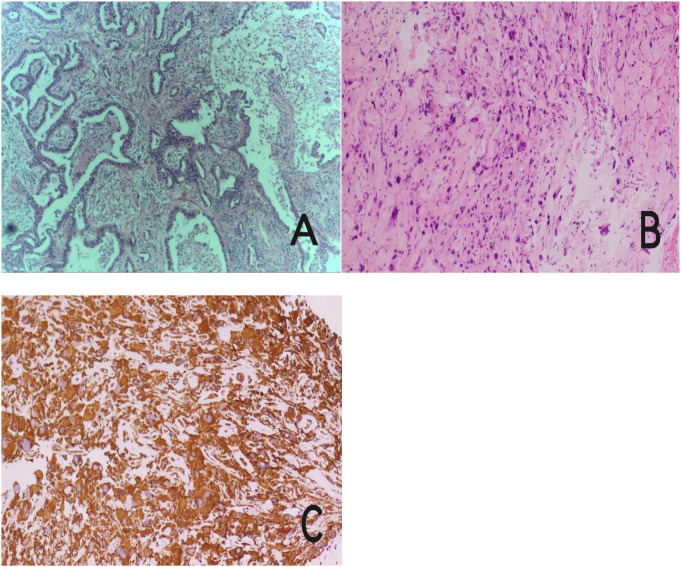
Histological morphology under light microscope. (**(A)** Surgical specimen at the initial diagnosis in 2022, lung adenocarcinoma, HE ×20. **(B)** Bronchoscopic neoplasm biopsy specimen from 2025, pulmonary giant cell carcinoma, HE ×100. **(C)** Biopsy specimen in 2025, immunohistochemistry for Vimentin ×100).

Three years ago, he underwent lobectomy of the right lower lobe and the posterior segment of the right upper lobe for lung adenocarcinoma (**Figures 1E, F**). Postoperative pathology revealed invasive adenocarcinoma (non-mucinous, Grade 2 moderately differentiated, acinar type in approximately 30%, papillary type in approximately 70%) (**Figure 2A**). Pathological pTNM stage was T4N1 (Stage IIIA). Immunohistochemical staining showed Ki-67 (+, 20%), CK7 (+), TTF-1 (+), mutant-type p53, Napsin A (+), CK5/6 (−), P40 (−), pan-CK (+), Vimentin (+), and PD-L1 (22C3) with TPS < 1%. The patient recovered well after surgery. Three cycles of chemotherapy with the GP regimen were administered at an outside hospital in January 2023. One year prior, re-examination of the chest CT scan due to hemoptysis indicated tumor recurrence. Genetic testing identified KRAS p.G12C (exon 2, variant allele frequency: 23.2%), PIK3CA p.H1047R (exon 21, variant allele frequency: 42.3%), and TP53 c.672 + 1G>T splice variant (variant allele frequency: 16.1%).

### 2.2 Physical examination

The thorax had no deformity, respiratory movement was symmetrical, and there was no sternal tenderness or subcutaneous emphysema. The patient’s abdominal breathing was normal. Both lungs were resonant on percussion, and vocal resonance was normal. Breath sounds decreased in the right lung, moist rales were heard, and there was no pleural friction rubbing. No obvious abnormalities were found in other examinations.

### 2.3 Auxiliary examinations

#### 2.3.1 Imaging examination

Chest CT (**Figures 1A, B**) revealed space-occupying lesions in the posterior segment of the right upper lobe and the dorsal segment of the right lower lobe.

#### 2.3.2 Laboratory tests

Blood routine showed WBC 10.8×10^9^/L↑, neutrophil percentage 77.7%↑, neutrophil count 8.42×10^9^/L↑, monocyte count 0.9×10^9^/L↑, lymphocyte percentage 11.6%↓, RBC 3.97×10¹²/L↓, Hb 114 g/L↓, HCT 35.7%↓, MCHC 320 g/L. Liver and kidney function and tumor markers (CEA, SCC, and NSE) were within normal ranges.

#### 2.3.3 Etiological examination

No cancer cells were found in sputum exfoliative cytology, and no bacterial or fungal growth was observed in the sputum culture.

### 2.4 Preliminary diagnosis

Malignant tumor of the right lung and obstructive pneumonia.

**Figure 3:**
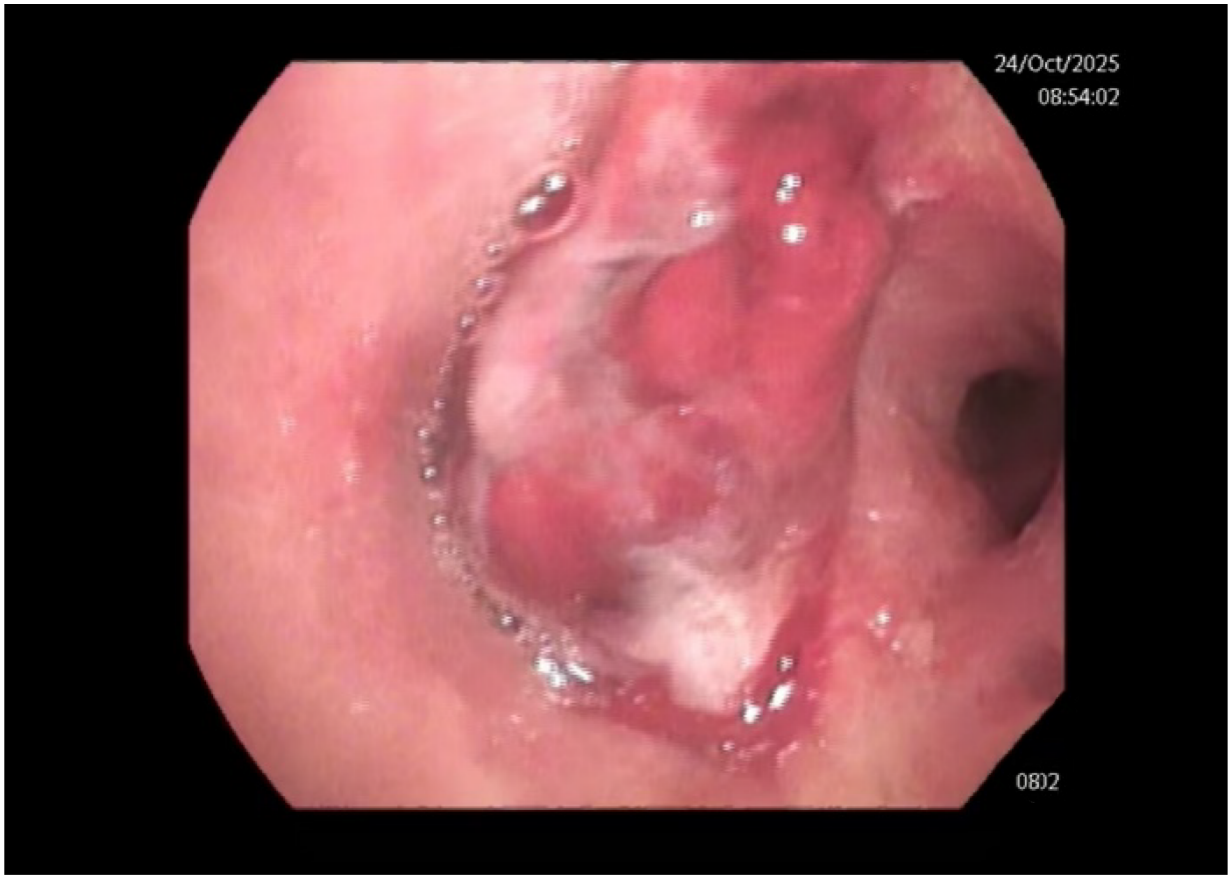
Fiberoptic bronchoscopic findings. Fiberoptic bronchoscopy performed after immunotherapy in 2026; A mass obstructing the lumen at the opening of the right intermediate bronchus, with irregular hyperplasia on the surface and marked luminal stenosis).

Fiberoptic bronchoscopy was performed for further diagnosis. Bronchoscopy (**Figure 3**) revealed a mass obstructing the lumen at the opening of the right intermediate bronchus, with irregular hyperplasia on the surface and marked luminal stenosis. Cryointerventional therapy was performed at the opening of the right intermediate bronchus to remove the mass, and the airway stenosis improved after surgery. No obvious abnormalities were observed in other lung. Pathological examination of the mass tissue showed (biopsy of the neoplasm at the opening of the right intermediate bronchus) combined with histochemistry and staining results consistent with non-specific non-small cell carcinoma with giant cell carcinoma morphology (**Figure 2B**).

### 2.5 Immunohistochemical staining

Results were as follows (**Figure 2C**) CK (AE1/AE3) (partially positive), TTF-1 (negative), vimentin (positive), Ki-67 (positive rate 30%), WT1 (negative), and calretinin (negative).

### 2.6 Genetic testing results

#### 2.6.1 Targeted panel

KRAS G12C mutation, PIK3CA mutation, TP53 mutation.

#### 2.6.2 Immunology panel

PD-L1 protein expression positive. Repeated genetic testing of the tissue samples revealed a persistent KRAS G12C mutation, and no other new driver gene mutations were detected.

### 2.7 Definitive diagnosis

PGCC (stage IV); obstructive pneumonia; pathological transformation after postoperative targeted therapy for lung adenocarcinoma.

The patient was in poor general condition on initial admission, complicated by obstructive pneumonia and elevated leukocytes, and was temporarily intolerant to combined chemotherapy. Palliative radiotherapy was refused by family members. Anti-infective therapy with piperacillin-tazobactam was administered for 1 week, after which infection indicators returned to normal and the patient’s condition stabilized. The patient and their family have explicitly declined curative resection and definitive local radiotherapy, making curative local treatment infeasible. Subsequently, immunotherapy with camrelizumab (200 mg, q3w) combined with anti-angiogenic therapy with endostar (15 mg, d1–14, q3w) was administered.

Currently, two treatment cycles have been completed. Re-examination of chest CT (**Figures 1C, D**) showed postoperative changes of the right lung, partially unclear local display of the right middle and lower lobes, high-density suture shadow in the right hilum, multiple patchy high-density shadows in the residual right lung; increased air content in both lungs with multiple cystic hyperlucency; multiple patchy high-density shadows in the left upper lobe; patent trachea and main bronchial openings, no enlarged mediastinal lymph nodes, and liquid density shadow in the right thoracic cavity. Baseline measurable target lesion in the right lung was 5.8 × 4.7 cm before camrelizumab combined with endostar treatment. After combination therapy, the lesion decreased to 2.1 × 1.7 cm. The tumor response evaluation strictly complied with RECIST version 1.1 criteria, with a maximum diameter reduction rate of approximately 63.8%, which met the diagnostic standard for partial response (PR).No obvious treatment-related adverse reactions were observed, and the patient was followed-up continuously.

## 3 Discussion

Straus and Schmidt first discovered PGCC in 1958. Through microscopic observation of lung tumors, they identified tumors composed of characteristic multinucleated giant cells, clearly distinguishing them from morphologically similar sarcomas and other tumors, and established them as independent pathological types of lung cancer (3). According to the 2015 WHO Classification of Tumors of the Lung, Pleura, Thymus, and Heart, PGCC is clearly classified as a subtype of pulmonary sarcomatoid carcinoma (1). Due to the lack of large-sample clinical data, the incidence of PGCC is difficult to accurately estimate. Using the US Surveillance, Epidemiology, and End Results (SEER) database, A study (4) analyzed lung cancer data from 1977 to 2010 and showed that the incidence of large-cell lung cancer was approximately one case per 100, 000 person, and the incidence of PGCC was even lower. The gold standard for the diagnosis of PGCC is histopathological examination, which can be combined with immunohistochemical staining (for CK, CK7, Vimentin, CK5/6, Ki-67, etc.) to improve diagnostic accuracy. Its pathological feature is that the tumor tissue is completely composed of highly atypical multinucleated or mononucleated tumor giant cells, often presenting as large necrotic masses without an obvious tissue structure. Microscopically, a large number of neutrophils and lymphocytes infiltration can be seen; tumor cells are large and multinucleated with bizarre morphology, and cell sizes differ by more than 5 times (5). The immunohistochemical features of PGCC are mostly partial positivity for epithelial markers (CK, pan-CK), diffuse positivity for mesenchymal marker (Vimentin), and negativity for lung adenocarcinoma-related markers (TTF-1, Napsin A, CK7) (6), which is highly consistent with the immunophenotype of the present case. Compared with other non-small cell lung cancers, PGCC has distinct clinical characteristics: it is more common in young males, some with a smoking history; mostly advanced stage (III/IV) at diagnosis; common symptoms including cough, hemoptysis, chest pain, and fever; a high incidence of distant metastasis; and a 5-year survival rate of only 5%–10%, which is significantly lower than that of other non-small cell lung cancer subtypes. Moreover, most PGCCs are pathological grade III/IV, and distant metastases is often present at the time of diagnosis (7). Currently, the mechanism of transformation from lung adenocarcinoma to PGCC is unclear. The treatment of such transformed lung cancer is mainly based on the standard regimen for PGCC: radical surgical resection is the first choice and the core method to achieve clinical cure, but most patients lose the opportunity for surgery at diagnosis; the efficacy of chemotherapy and radiotherapy is limited; immunotherapy has a significant effect on PGCC, and has become an important treatment option for advanced patients due to the frequent high PD-L1 protein expression (8).

*KRAS* mutations are important carcinogenic drivers of non-small cell lung cancer (NSCLC), among which the *G12C* mutation is the most common subtype (9). *KRAS* gene mutations continuously activate downstream signaling pathways, leading to uncontrolled cell proliferation, malignant transformation, and further promotion of tumor occurrence and development. The histological subtype transformation of NSCLC is a rare mechanism of targeted therapy resistance, mostly observed in EGFR-mutant lung adenocarcinoma transforming to small cell lung cancer or squamous cell carcinoma, whereas transformation to PGCC has rarely been reported (10).The present case was a lung adenocarcinoma with a *KRAS G12C* mutation that progressed after 1 year of sotorasib-targeted therapy and transformed into pulmonary giant cell carcinoma. Based on the clinical data of this case and the related literature, we put forward two hypothetical mechanisms that may potentially explain this transformation event:

### 3.1 Treatment pressure-induced tumor cell dedifferentiation

One study (11)found that lung tumors may develop drug resistance, leading to changes in the tumor pathological type under the pressure of chemotherapy, targeted therapy, immunotherapy, or radiotherapy. Sotorasib, a *KRAS G12C* inhibitor, exerts its antitumor effects by inhibiting the activity of mutant *KRAS* proteins. However, long-term treatment can induce phenotypic conversion of tumor cells. In this case, the patient experienced disease progression following prolonged sotorasib treatment, accompanied by subsequent histological subtype alteration. This chronological sequence raises a hypothesis that the selective therapeutic pressure induced by long-term sotorasib administration may serve as one potential contributing factor associated with the transformation from lung adenocarcinoma to pulmonary giant cell carcinoma, without confirming a definitive causal relationship. Another research (12) found that AT1-like cells isolated from residual lesions of lung adenocarcinoma after targeted therapy have high growth potential, and lineage tracing shows that these cells are plastic and can transdifferentiate into other lung cancer cell states, serving as a “safe harbor” for lung adenocarcinoma cells under therapeutic pressure until acquired resistance develops. Residual lesions in lung adenocarcinoma after *KRAS*-targeted therapy are mainly composed of AT1-like cells capable of restarting tumorigenesis. These cells are in an intermediate state of dedifferentiation of lung adenocarcinoma cells under drug pressure, have lost the characteristics of the original adenocarcinoma (such as TTF-1 positivity), and show more primitive and stronger plasticity. It has been speculated that the plasticity of AT1-like cancer cells confers them with the ability to transform into pulmonary giant cell carcinoma. The initial surgical specimen of this patient was TTF-1 (+), and the transformed biopsy specimen was TTF-1 (-). This result supports the speculation of tumor cell dedifferentiation, suggesting that the plasticity of AT1-like cells may be the cytological basis for their transformation to giant cell carcinoma.

### 3.2 Activation of heterogeneity of cancer stem cells

There are a small number of cancer stem cells with multidirectional differentiation potential in lung cancer tissues. Targeted therapy kills only drug-sensitive tumor cells, whereas cancer stem cells can survive under drug pressure and further differentiate into new pathological subtypes (13). It has been speculated that PGCC and lung adenocarcinoma may have originated from the same cancer stem cell population. It is theoretically possible that upon acquiring resistance to KRAS inhibitors, such stem cell populations could undergo divergent differentiation toward a giant cell carcinoma phenotype, which may offer one speculative explanation for the observed histological transformation; this proposed pathway remains unproven and requires further experimental verification.

### 3.3 Study limitations

This study is a retrospective single-case report. Due to the limitations of clinical medical record preservation, some detailed historical treatment information and dynamic monitoring data of the patient were not completely acquired. In addition, only a single case was included in this study, which lacks large-sample validation. Therefore, the relevant conclusions need to be further verified by more clinical studies.

## 4 Clinical implications

The diagnosis and treatment process in this case provides an important reference for the clinical management of lung cancer after targeted therapy resistance. The core clinical implications of this case study are as follows:

### 4.1 Emphasis on the clinical value of re-biopsy after targeted therapy

After developing resistance to targeted therapy, tumors may undergo pathological subtype transformations or changes in molecular characteristics. Repeat biopsy is a key step in clarifying the mechanism of resistance. If a fiberoptic bronchoscopic biopsy was not performed, treatment would have been continued based on lung adenocarcinoma resistance, delaying the optimal treatment opportunity. Therefore, for lung cancer patients with disease progression after targeted therapy, active re-tissue biopsy should be pursued and individualized treatment plans should be formulated in combination with pathological examination and genetic testing results. Notably, many advanced lung cancer patients cannot tolerate highly invasive biopsy procedures. For patients complicated with malignant pleural effusion, repeated minimally invasive cytological sampling via cell block preparation is a low-trauma alternative. Wang et al. (14) confirmed that repeated percutaneous closed pleural brushing (CPBR) combined with cell block technology achieves a cumulative positive diagnostic rate of 89% after two samplings; this minimally invasive repeated sampling approach can harvest sufficient tumor cells to support subsequent immunohistochemical staining and molecular profiling.

### 4.2 Rational selection of treatment regimens for advanced PGCC

Advanced PGCC is less sensitive to conventional chemotherapy, whereas immunotherapy combined with anti-angiogenic therapy shows definite therapeutic efficacy. The tumor lesion in this patient significantly reduced after treatment with camrelizumab combined with endostar, which is consistent with the therapeutic effect reported in the related literature (15). In addition, some patients with PGCC may carry specific driver gene mutations, such as *KRAS* and *ALK*, and targeted therapy can still be used as a treatment option for such patients (16), which requires comprehensive judgment combined with genetic testing results.

### 4.3 Emphasis on long-term regular follow-up after targeted therapy for lung adenocarcinoma

Even if the initial efficacy of targeted therapy is significant, regular reexamination is required for patients with lung adenocarcinoma to detect disease progression in a timely manner. The resistance patterns of patients with *KRAS G12C*-mutant lung adenocarcinomas are diverse. In addition to the pathological subtype transformation, new gene mutations and bypass signal activation may occur. Regular long-term follow-ups help achieve early intervention in disease progression and improve the clinical prognosis of patients.

Targeted therapy resistance is a major challenge in clinical management. The core lies that Tumor cells can evade drug inhibition through various molecular mechanisms during repeated treatments, leading to treatment failure and disease progression. The key to solving this dilemma is to actively perform re-tissue biopsy for patients with resistance to targeted therapy and formulate individualized subsequent treatment plans combined with pathological classification and genetic testing results. Long-term regular follow-up is crucial to capture timely signals of disease progression, achieve early clinical intervention, and ultimately improve survival prognosis.

This study was supported by the Jilin Provincial Science and Technology Development Plan Project (Grant No. YDZJ202501ZYTS786). All authors have read and approved the content and had no conflicts of interest. There are no ethical/legal conflicts involved in the article. The authors declare that the research was conducted in the absence of any commercial or financial relationships that could be construed as a potential conflict of interest.

## Timeline

**Table 1:** Timeline recording key clinical events, imaging, pathological and genetic examination results, as well as treatment procedures of the patient with KRAS G12C-mutant lung adenocarcinoma transforming to pulmonary giant cell carcinoma after sotorasib resistance.

Time node	Core clinical events & test results
2022.11	• Diagnosed with right lung adenocarcinoma; received lobectomy of right lower lobe + posterior segment of right upper lobe • Postoperative pathology: Invasive non-mucinous adenocarcinoma (Grade 2), IHC TTF-1 (+) • Chest CT: Space-occupying lesion in right middle lobe • Uncomplicated postoperative recovery
2024 (2 years post-surgery)	• Tumor recurrence confirmed by chest CT (for hemoptysis) • Genetic test: KRAS G12C mutation (exon 2) detected • Initiated sotorasib (KRAS G12C inhibitor) targeted therapy
2025.10	• Admitted for fever, cough, asthma (>10 days) • Tests: Blood routine showed infection + mild anemia; chest CT found new space-occupying lesions in right lung; normal tumor markers/liver-kidney function • Fiberoptic bronchoscopy: Cryoablation for obstructive mass in right intermediate bronchus • Pathological confirmation: Transformed to PGCC • Definitive diagnosis: PGCC (Stage IV) + obstructive pneumonia • Piperacillin-tazobactam anti-infection for 1 week, condition stabilized • Started combined therapy: Camrelizumab (200mg, q3w) + Endostar (15mg, d1-14, q3w)
2026.1	• Completed 2 cycles of immunotherapy + anti-angiogenic therapy • Chest CT: Tumor shrank to 2.1cm×1.7cm, no mediastinal lymphadenectasis • Efficacy: Partial Response (PR), no obvious adverse reactions • Under continuous follow-up

## Patient perspective

The patient reported significant relief of fever and asthma after anti-infective and combined immunotherapy, though a severe cough persisted during the two cycles of intervention. The patient maintained good treatment compliance throughout the course and had no obvious subjective discomfort such as fatigue or nausea. The patient expressed a clear understanding of the disease transformation and treatment plan, and was willing to continue follow-up and subsequent clinical treatment.

## Data Availability

The original contributions presented in the study are included in the article/supplementary material. Further inquiries can be directed to the corresponding author.
